# An Unusual Case of Wernicke's Encephalopathy in a Child

**DOI:** 10.7759/cureus.27260

**Published:** 2022-07-25

**Authors:** Samuel O Nwaobi, Denisia N Thomas, Amaka C Ugoh

**Affiliations:** 1 Family Medicine, Piedmont Columbus Regional-Midtown, Columbus, USA; 2 Family Medicine, Edward Via College of Osteopathic Medicine, Auburn, USA; 3 Family Medicine, University of Benin Teaching Hospital, Benin, NGA

**Keywords:** neurology and critical care, newborn and child health, nutritional deficiency, beriberi, wernicke encephalopathy, non-alcoholic wernicke's encephalopathy

## Abstract

Wernicke’s encephalopathy (WE) is a manifestation of thiamine deficiency. The majority of affected patients are alcoholics and are adults. Often, clinicians fail to recognize that WE can also be found in non-alcoholic patients at risk for thiamine deficiency. Sometimes patients may not present with all the classic features, or the individual clinical signs may be treated as single problems and not a constellation of signs that form a diagnosis of WE. We present a unique case of a four-year-old male with a past medical history of food aversion who presented with intractable vomiting and weakness. The patient’s clinical features showed signs of severe dehydration and weight loss. His clinical state subsequently progressed to having ophthalmoplegia and gait ataxia. Brain MRI demonstrated mamillary body changes, and serum thiamine level was significantly below the normal limit. Based on the patient’s clinical assessment, deficient serum thiamine, and MRI findings, WE was diagnosed. The patient was evaluated by Pediatric Neurology and started on treatment with high dose IV thiamine. He showed an excellent response to thiamine treatment and had a significant resolution in his symptoms before discharge.

## Introduction

Wernicke’s encephalopathy (WE) is a rare neurologic emergency caused by severe thiamine deficiency, which, if not treated early, can lead to complications such as confusion, loss of mentation, coma, and death [1]. The diagnosis of WE is clinical and requires two of the following to make a diagnosis in alcoholics; ocular manifestations, cerebellar dysfunction, and an altered mental state or mild memory impairment [2]. Though lab and imaging studies such as low serum thiamine levels and changes in the mamillary body on imaging may aid the diagnosis, these findings alone are not diagnostic. Although commonly seen in alcoholics, WE can also be seen in patients with anorexia nervosa, patients undergoing dialysis, prolonged parenteral nutrition, prolonged fasting, and bariatric surgery. The prevalence of WE is not well known; most affected patients are alcoholic adults, and the prevalence (based on autopsy studies) is estimated to be about 0.4%-2.8%, accounting for about 1.3% of all autopsies. One of the major barriers to early diagnosis is the low index of suspicion in non-alcoholic patients who do not have the complete classical triad on presentation, including altered mental status, ataxic gait, and ophthalmoplegia [3]. The treatment of WE is relatively easy and effective. Once diagnosed and treated early, the prognosis is good. If a case is misdiagnosed, worsening neurologic status and ultimately death may occur.

## Case presentation

This is a case of a four-year-old Caucasian male with a medical history of a selective eating disorder who presented to the Pediatric Emergency Department (ED) with his mother complaining of vomiting and difficulty tolerating oral intake of four days duration. For the past six months, the patient had difficulty swallowing, decreased appetite, and the subsequent development of food aversion. Sometimes, he would gag or cry if he saw his mother or siblings eating. He occasionally would eat small meal portions and drink Kool-Aid. The patient had been evaluated by an ear, nose, and throat (ENT) specialist who diagnosed him with adenoid hypertrophy but did not think this was a significant cause of his food aversion. The ENT provided the patient with antibiotics and steroids with no plan for surgery. At the onset of vomiting, the patient was brought to the ED by his mother and was treated with IV fluids and ondansetron. He was discharged home and instructed to follow up with his primary care provider (PCP). Two days later, he returned to the Pediatric ED after visiting his PCP due to worsening vomiting, decreased oral intake, dehydration, and generalized weakness. The patient’s mother denied fever, diarrhea, or respiratory symptoms and provided an unremarkable birth history. The patient was up to date on vaccinations.

**Figure 1:**
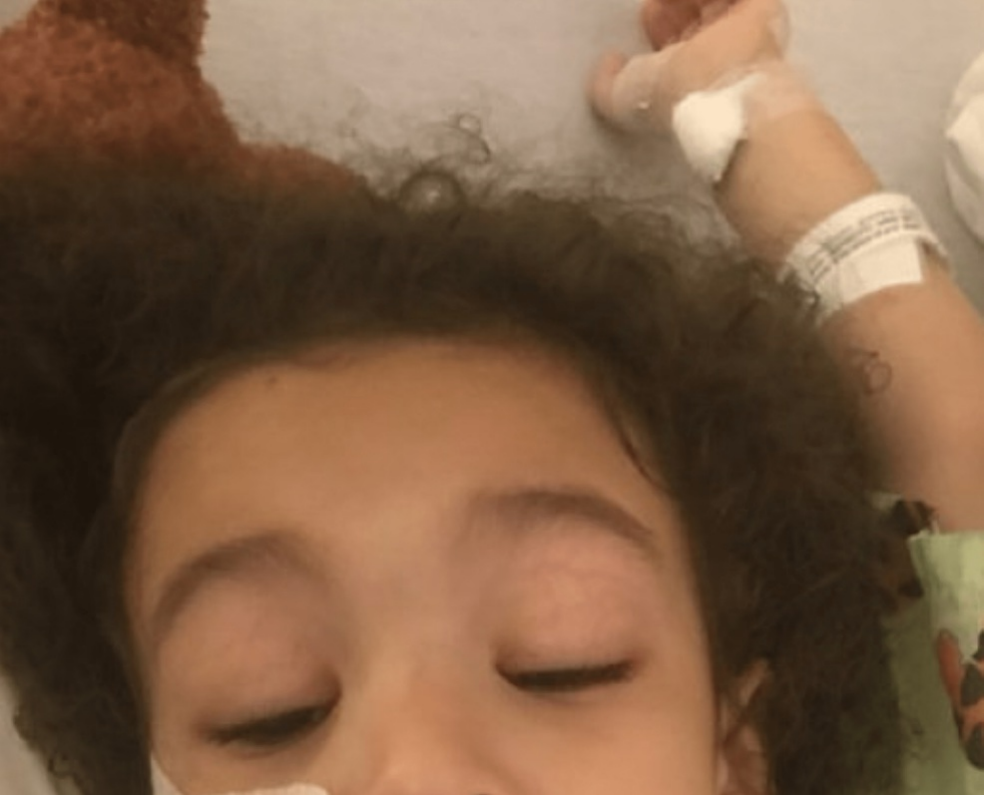
Complete ophthalmoplegia with ptosis.

**Figure 2:**
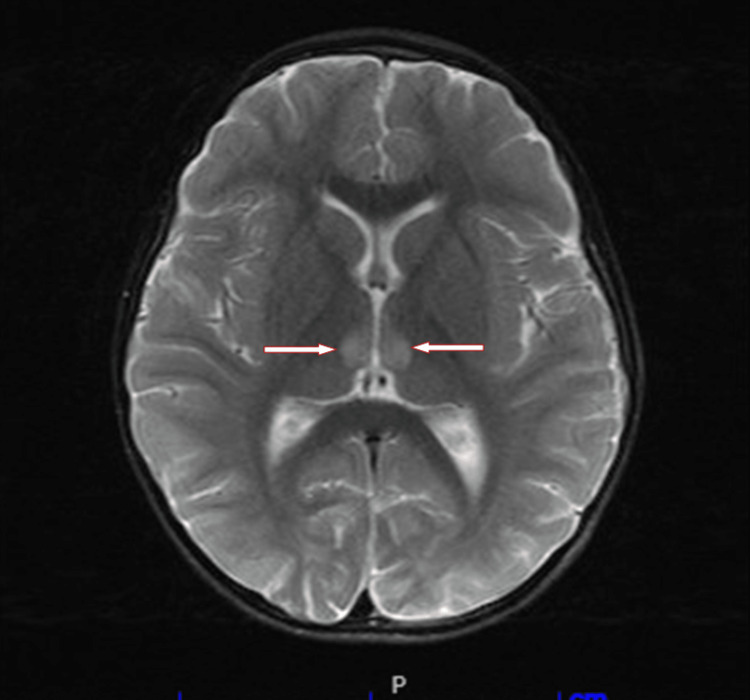
Brain MRI showing abnormal hyperintense lesion in the mammillary bodies.

On a physical exam, the patient was lethargic, with sunken eyes, dried mucous membranes, and angular stomatitis. Capillary refill time was about five seconds. He weighed 13.3 kg and was in the first percentile of weight (based on the Centers for Disease Control and Prevention weight-for-age percentiles) on presentation. On day 1 of admission, the laboratory workup showed the patient to have an elevation in the anion gap with metabolic acidosis with pH 7.13 /HCO3 6.0/PCO2 18.4, Na+ 142, K+ 5.1, and Cl- 111. Glucose was 125 mg/dL and anion gap 25 mmol/L. Urinalysis was positive for ketones. Leukocytosis was noted with a white blood cell (WBC) of 33.29 103/µL (reference range: 4.00-10.50) with concerns for sepsis of unknown source; he was started on IV ceftriaxone while blood cultures were pending. Lactate levels and chest radiograph were unremarkable. The patient was started on 5% dextrose (D5) in 0.45% normal saline (NS) and then changed to 0.45% NS once glucose levels were normal. On day 2, it was noted that the anion gap had closed. However, bicarbonate levels remained below 16 mmol/L despite rehydration and IV sodium bicarbonate treatment. Sodium, potassium, and chloride levels were all within normal limits. Anion gap was 17 mmol/mL (reference range: 10-17 mmol/mL). Extensive metabolic testing to assess for renal tubular acidosis and diabetes was done following consultation from the Endocrinologist on call. The workup included serum methylmalonic acid assay, urine pH, and amino acids. Further testing included insulin growth factor to assess growth hormone deficiency, HbA1c to rule out diabetes mellitus and repeat lactic acid levels to assess lactic acidosis. The results were normal. Blood culture sets were negative after 48 h. On day 3, the patient became more lethargic with bilateral ptosis (Figure 1), abnormal ocular abduction, upgaze palsy, and difficulty walking. CT of the head was unremarkable. However, an MRI of the brain showed that the medial aspects of the thalamus, mammillary bodies, and periaqueductal region appeared hyperintense in the diffusion-weighted images (Figure 2).

Neurology was consulted and recommended thiamine and vitamin B12 level testing. The patient was started on IV thiamine 100 mg daily and given vitamin B12. His thiamine level was significantly low, <6 nmol/L (reference range: 8-30 nmol/L). After three days of thiamine supplementation and parenteral nutrition, his neurologic symptoms and appetite improved. He also had improvement in his bicarbonate levels and other electrolytes. By day 9 of hospitalization, the patient was in the fifth percentile for weight. He was playful and able to ambulate appropriately. He was discharged with a scheduled appointment with his pediatrician and a prescription for oral thiamine. The patient had a routine follow-up four weeks post-discharge and was noted to have a normal neurological examination. He was at the tenth percentile for weight, which increased from the first percentile at the time of initial presentation.

## Discussion

Thiamine, also known as vitamin B1, is a water-soluble vitamin absorbed into the blood from the small intestine. Small amounts of thiamine are stored in the liver, heart, kidney, and brain for a short duration. It has a short half-life of 14-18 days; therefore, regular dietary intake is necessary. Foods rich in thiamine include whole grains, brown rice, pork, poultry, soybean, nuts, dried beans, peas, and fortified or enriched grains such as cereals, infant formulas, and bread. Thiamine serves as a cofactor for many enzymes during the metabolism of glucose, proteins, and lipids [4]. Thiamine deficiency can result in a multitude of complications, including cardiomyopathy (wet beriberi), peripheral neuropathy (dry beriberi), psychosis, altered mental status, and WE [5].

There is limited data on WE in the pediatric population, with most cases having a mean age of 40. Most cases of WE go undiagnosed, with an estimated 80% of patients being misdiagnosed [6]. Vasconcelos et al. found that only a few cases of WE in children initially present with the classic triad, and about 41.9% of cases are diagnosed at postmortem examination [7]. It is important to suspect WE as a possible diagnosis in all patients, both children, and adults, with a history of poor nutritional intake or nutritional losses. Our patient had a history of selective eating disorder, adenoid hypertrophy, and possibly an acute viral infection associated with intractable vomiting. This combination of long-term limited nutritional intake and sudden increased nutritional losses due to vomiting may have led to the patient developing thiamine deficiency. The patient was briefly administered IV dextrose, which may have unmasked an underlying thiamine deficiency and exacerbated his symptoms. Thiamine is an essential cofactor in carbohydrate metabolism. A study by Smith et al., indicated that dextrose-containing fluids could increase thiamine requirements and may worsen symptoms of WE [8]. The Wernicke-Korsakoff syndrome is a disorder composed of two different diseases, each term describing a different stage. WE is an acute illness necessitating emergent treatment with thiamine to prevent neurologic derangement and death. In the second stage, Korsakoff syndrome (KS) is a chronic disease characterized by neuropsychiatric symptoms that are brought on as a complication and further consequence of WE. Early detection is key to preventing the progression of WE into KS [9]. MRI is becoming a useful adjunct in the early diagnosis of WE and may increase the early detection of cases. Common MRI findings include symmetric T2 hyperintensities in the dorsal medial thalamus, mammillary bodies, periaqueductal gray matter, and the tectal plate [10].

## Conclusions

We report a case of a four-year-old Caucasian male with a history of poor dietary intake, dehydration, and intractable vomiting who developed WE at an unusual age. The presence of a nutritional insufficiency such as thiamine deficiency should always be considered in patients with a history of poor oral intake and intractable vomiting. The administration of dextrose-containing fluids should be avoided if WE is suspected. The fact that our patient was a child and initially had metabolic derangements made the early diagnosis of WE difficult. Identifying clinical signs and using imaging modalities such as MRI is important for early WE diagnosis. Diagnosis and early treatment of thiamine deficiency may decrease progression to WE and allow for a better prognosis.
